# Sex-specific signatures of gut microbiota and systemic inflammation in patients with urolithiasis: a cross-sectional study

**DOI:** 10.3389/fcimb.2026.1835752

**Published:** 2026-06-26

**Authors:** Hao Chen, Fengyi Tian, Shuqing Sheng, Mingrui Wang, Xiangcheng Zhan, Yuchen Gao, Bowen Chen, Yunze Dong, Shuai Liu, Yanhua Chen, Xudong Yao, Tiancheng Xie, Zhuifeng Guo, Yunfei Xu

**Affiliations:** 1 Department of Urology, Shanghai Tenth People’s Hospital, School of Medicine, Tongji University, Shanghai, China; 2 Department of Urology, Minhang Hospital, Fudan University, Shanghai, China

**Keywords:** 16S rRNA, disease phenotyping, gut microbiome, sex differences, systemic inflammation, urolithiasis

## Abstract

**Background:**

Urolithiasis is a globally prevalent disease with a distinct male predominance; however, the pathophysiological heterogeneity within diagnosed cohorts remains underexplored. This study delineates the sex-specific signatures of gut microbiota and systemic inflammation in urolithiasis patients to inform sex-stratified management.

**Methods:**

This cross-sectional study enrolled 60 urolithiasis patients (40 males, 20 females). Systemic inflammatory cytokines were quantified via peripheral blood assays, and gut microbiota was profiled using 16S rRNA sequencing. Data were integrated to evaluate microbiome-immune-metabolic associations.

**Results:**

Baseline demographics and routine biochemical parameters were comparable between sexes. Male patients exhibited significantly elevated peripheral levels of pro-inflammatory cytokines, including IL-5, IL-17A, IFN-α, IL-12P70, and IFN-γ (P < 0.05). Beta-diversity analysis revealed no significant difference in the overall gut microbial community structures between sexes (P = 0.484). LEfSe analysis identified a significant enrichment of *Akkermansia* and *Holdemanella* in females, whereas *Mogibacterium* was notably enriched in males. Crucially, *Mogibacterium* abundance positively correlated with IL-17A and IL-12P70 levels (P < 0.05). Functional potential profiling indicated enhanced predicted capacities for secondary metabolite biosynthesis and lipid metabolism in the female cohort.

**Conclusion:**

Our findings highlight significant sex-associated differences in gut microecology and systemic immune profiles within urolithiasis patients. The proinflammatory axis associated with male patients and the enhanced predicted metabolic capacities observed in female patients emphasize the potential value of exploring sex-tailored preventive and therapeutic interventions.

## 1 Introduction

Urolithiasis is a major global health issue, with prevalence rates ranging from 2% to 20% worldwide (Raheem et al., 2017; Kachkoul et al., 2023). This widespread incidence translates into a massive clinical and economic burden (Lang et al., 2022). Epidemiologically, men have historically been over twice as likely to be affected as women (Xu et al., 2022; Ferraro et al., 2023; Tan et al., 2024). Recent shifts in modern lifestyles, dietary changes such as increased consumption of sugar-sweetened beverages and growing waist circumferences, have been shown to drive a more lithogenic urinary profile (Ferraro et al., 2023). These lifestyle risk factors impact urolithiasis by altering urine chemistries, notably by reducing urine volume and pH while increasing the excretion of oxalate and sodium. While evolving exposure to these factors has begun to narrow the traditional sex gap by accelerating stone incidence in women, males still exhibit a much higher overall susceptibility and long-term recurrence rate (Lin et al., 2020; Skolarikos et al., 2024). Consequently, elucidating the underlying pathophysiological mechanisms is crucial for developing sex-stratified preventive strategies.

This sex disparity is driven by a multifactorial interplay of hormonal, anatomical, metabolic, and behavioral factors. Hormonally, estrogen inhibits calcium oxalate crystallization, protecting reproductive-aged women, whereas androgens promote lithogenesis (Liang et al., 2014; Ferraro et al., 2016; Prochaska et al., 2018). Anatomically, the longer, more tortuous male urethra delays stone passage, whereas the shorter female urethra facilitates spontaneous clearance. Moreover, metabolic and lifestyle risks, including obesity, dyslipidemia, high-protein diets, and occupational dehydration, further elevate male susceptibility (Taylor et al., 2005; Carbone et al., 2018). Currently, clinical management relies on conservative medical expulsive therapy, extracorporeal shock wave lithotripsy (ESWL), and surgical interventions (Pearle et al., 2026). However, these classical paradigms cannot fully explain the complex pathogenesis and recurrence, prompting investigations into alternative mechanisms (Pei et al., 2025).

Investigations into the “Gut-Kidney Axis” underscore the critical role of dysbiosis in urolithiasis (Adak and Khan, 2019; Ticinesi et al., 2020). While previous research predominantly focused on the direct litholytic capacity of specific taxa like *Oxalobacter formigenes* (Siva et al., 2009; Ticinesi et al., 2019), recent studies highlight how dysbiosis-mediated systemic inflammation fundamentally alters the local urinary microenvironment (Nikolic-Paterson et al., 2014; Miller et al., 2019; Dominguez-Gutierrez et al., 2020). Crucially, broader studies on urolithiasis pathogenesis have largely overlooked how intrinsic sex differences contribute to distinct phenotypic profiles within diagnosed cohorts.

Gut microecology and systemic immunity differ notably between the sexes. Women generally harbor a metabolically protective microbiome and stronger immune tolerance, while men tend to lean toward a pro-inflammatory state (Kim et al., 2020). Consequently, male and female patients can develop entirely different systemic inflammatory environments, even when facing the exact same crystal burden (Tang et al., 2025). Despite these known differences, clinical data characterizing sex-specific phenotypes in active urolithiasis remain scarce. We conducted this study to fill that gap by analyzing a strictly diagnosed cohort. We combined 16S rRNA gene sequencing with peripheral blood cytokine profiling to explicitly evaluate the associations between signature bacteria and systemic inflammation in both sexes. Ultimately, these insights provide an exploratory foundation for investigating sex-stratified strategies in urolithiasis management.

## 2 Materials and methods

### 2.1 Study design and cohort definition

This cross-sectional study was conducted in accordance with the Declaration of Helsinki and was approved by the Ethics Committee of Shanghai Tenth People’s Hospital Affiliated to Tongji University (Approval ID: 21K105). Written informed consent was obtained from all participants prior to enrollment. A total of 60 patients with urolithiasis (40 males and 20 females) admitted to the hospital between October 2022 and May 2024 were prospectively enrolled for gut microbiota analysis.

The inclusion criteria were defined as follows: (1) age ≥ 18 years; (2) urolithiasis definitively diagnosed via color Doppler ultrasound or computed tomography (CT) imaging; and (3) for female participants, either a completely postmenopausal status (natural amenorrhea for ≥ 12 consecutive months) or the presence of regular menstrual cycles (28 ± 7 days). To rigorously control for the confounding effects of cyclical hormonal fluctuations on systemic inflammation and the gut microbiome, premenopausal women were exclusively sampled during the early follicular phase (days 2–5 of the menstrual cycle), a period characterized by baseline nadirs in estrogen and progesterone levels.

Patients were excluded if they met any of the following criteria: (1) a history of or currently active gastrointestinal diseases; (2) systemic administration of antibiotics or probiotics within the preceding three months; or (3) underlying endocrine or hormone-related disorders, current pregnancy or lactation, or recent use of hormonal medications.

### 2.2 Clinical assessment and biological sample collection

Upon admission, comprehensive demographic and clinical data, including age, sex, body mass index (BMI), stone characteristics (location and dimensions based on imaging), and comorbidities (e.g., hypertension, diabetes mellitus), were systematically recorded.

Prior to any therapeutic intervention, fasting venous blood and first-morning urine samples were procured from all patients. These samples were immediately processed by the institutional clinical laboratory for hematological profiling (including complete blood counts, hepatic and renal function panels, and C-reactive protein), urological cytology, and standard biochemical analyses. The standard clinical reference ranges for all routine parameters and inflammatory cytokines were established and validated by our institutional central laboratory. Concurrently, midstream clean-catch urine samples were collected for bacterial and fungal culturing and identification. To evaluate the systemic inflammatory milieu, serum was isolated from the venous blood, and the concentrations of key inflammatory cytokines (IL-2, IL-4, IL-5, IL-6, IL-17A, IL-12P70, IFN-α, and IFN-γ) were quantitatively assessed using a Cytometric Bead Array (CBA) or Enzyme-Linked Immunosorbent Assay (ELISA), strictly adhering to the manufacturers’ standardized protocols.

Additionally, fresh fecal samples were collected from all participants in sterile containers immediately upon admission. To preserve microbial community integrity and prevent structural alterations, the samples were rapidly transported on ice to the laboratory and cryopreserved at -80 °C until total microbial genomic DNA extraction.

### 2.3 16S rRNA gene sequencing and microbiome analysis

#### 2.3.1 DNA extraction and PCR amplification

Total microbial genomic DNA was extracted from the fecal aliquots utilizing the PF Mag-Bind Stool DNA Kit (Omega Bio-tek, Norcross, GA, USA). DNA integrity and purity were verified via agarose gel electrophoresis and a NanoDrop^®^ ND-2000 spectrophotometer (Thermo Scientific Inc., USA). To rigorously monitor for potential environmental and reagent contamination, negative controls (blank extraction buffers) were included during both the DNA extraction and amplification procedures. The hypervariable V4 region of the bacterial 16S rRNA gene was amplified using the universal primer pair 515F (5’-GTGCCAGCMGCCGCGG-3’) and 806R (5’-GGACTACHVGGTWTCTAAT-3’) on an ABI GeneAmp^®^ 9700 PCR thermal cycler (Applied Biosystems, CA, USA). All amplification reactions were performed in triplicate to ensure reproducibility, and the resulting PCR amplicons were extracted and purified from a 2% agarose gel.

#### 2.3.2 Illumina MiSeq sequencing

The purified amplicons were quantified, pooled in equimolar concentrations, and subjected to paired-end sequencing on the Illumina PE300 platform (Illumina, San Diego, CA, USA) following the standard operating protocols of Majorbio Bio-Pharm Technology Co., Ltd. (Shanghai, China).

#### 2.3.3 Bioinformatics and amplicon sequence processing

Raw paired-end sequencing reads (PE300) were demultiplexed and quality-filtered using fastp (v0.19.6) (Chen et al., 2018). The quality-control criteria were applied as follows: reads were truncated at any site receiving an average quality score of < 20 over a 50-bp sliding window, and truncated reads shorter than 50 bp or those containing ambiguous characters (N bases) were discarded. Paired-end reads were subsequently merged into longer sequences using FLASH (v1.2.7) (Magoč and Salzberg, 2011), requiring a minimum overlap length of 10 bp and allowing a maximum mismatch ratio of 0.2 within the overlap region.

The DADA2 plugin (Callahan et al., 2016) within the QIIME 2 pipeline (Bolyen et al., 2019) was employed to denoise the high-quality optimized sequences, utilizing sample-specific error models to generate single-nucleotide resolution Amplicon Sequence Variants (ASVs). In total, 4,704,589 raw reads were generated across the entire cohort, and 2,664 ASVs were successfully identified following the denoising and chimera removal pipeline. To mitigate the confounding effects of uneven sequencing depth on downstream microbiome diversity analyses, all samples were randomly rarefied to the minimum valid sequence count obtained from a single sample across the cohort (27,603 sequences), resulting in a final standardized count of 2,546 ASVs for downstream community analysis. This approach effectively normalized the data while maintaining an average Good’s coverage of > 99.0%. Taxonomic assignment of the ASVs was performed using the Naive Bayes consensus classifier in QIIME 2 against the SILVA 16S rRNA database (v138) with a confidence threshold of 0.7. Metagenomic functional profiles were predicted from the ASV representative sequences using PICRUSt2 (Phylogenetic Investigation of Communities by Reconstruction of Unobserved States) (Douglas et al., 2020). All downstream bioinformatics analyses were executed on the Majorbio Cloud Platform. Alpha diversity indices (Chao1, ACE, Shannon, and Good’s coverage indices) at the Amplicon Sequence Variant (ASV) level and rarefaction curves were calculated using Mothur (v1.30) (Schloss et al., 2009). Beta diversity was visualized using Principal Coordinate Analysis (PCoA) based on the Bray-Curtis distance matrix at the ASV level via the Vegan package (v2.4.3) to assess structural differences between microbial communities. Between-group structural differences were evaluated using permutational multivariate analysis of variance (PERMANOVA/Adonis) with 9,999 permutations. Additionally, spatial separations along the primary principal coordinate axes (PC1 and PC2) were assessed using the Wilcoxon rank-sum test. Linear Discriminant Analysis Effect Size (LEfSe) was utilized to identify discriminative bacterial taxa (from phylum to genus) significantly enriched between groups, applying an LDA score threshold of > 2.0 and P < 0.05.

### 2.4 Statistical analysis

All statistical evaluations were performed using R software (version 4.3.1). Normally distributed continuous variables were presented as mean ± standard deviation, while non-normally distributed data were reported as medians with ranges (maximum and minimum values). Categorical variables were expressed as frequencies and percentages and compared using the Chi-square test. Measurement data were compared using the t-test or Wilcoxon rank-sum test. For two-group comparative analyses, P-values were adjusted using the Benjamini-Hochberg False Discovery Rate (FDR) method. A two-tailed P-value < 0.05 was considered statistically significant.

## 3 Results

### 3.1 Clinical characteristics and sex-specific systemic inflammatory profiles

A total of 60 patients with urolithiasis were enrolled, comprising 40 males and 20 females. As detailed in **Table 1**, baseline demographic characteristics were comparable between the two cohorts. The mean ages for the male and female groups were 61.0 and 64.5 years, respectively, demonstrating no statistically significant difference. Among the 20 female participants, 17 were postmenopausal, while the remaining three were premenopausal with regular menstrual cycles. The mean Body Mass Index (BMI) values were 25.4 ± 3.4 kg/m² for males and 25.2 ± 4.1 kg/m² for females. The mean BMI values of both cohorts fell into the overweight category (BMI ≥ 24.0 kg/m²) according to the Working Group on Obesity in China guidelines. Furthermore, no statistically significant differences were observed between male and female patients regarding the prevalence of concurrent comorbidities, including diabetes mellitus (20.0% vs. 15.0%), hypertension (32.5% vs. 50.0%), and urinary tract infections (25.0% vs. 40.0%) (all P > 0.05). Stone characteristics, including anatomical location, size, and composition, also did not differ significantly between the groups.

**Table 1:** Clinical and laboratory characteristics of patients with urolithiasis stratified by sex.

Parameters	Reference range	Male	Female	P. value
		(N=40)	(N=20)	
Age (year)		61.0 (34.0, 78.0)	64.5 (38.0, 87.0)	0.335^a^
Diabetes		8 (20.0%)	3 (15.0%)	0.736^b^
Hypertension		13 (32.5%)	10 (50.0%)	0.189^c^
BMI (kg/m2)		25.4 (±3.4)	25.2 (±4.1)	0.880^d^
Infection		10 (25.0%)	8 (40.0%)	0.232^c^
Position				0.938^b^
Left		17 (42.5%)	10 (50.0%)	
Right		9 (22.5%)	4 (20.0%)	
Both		14 (35.0%)	6 (30.0%)	
Stone size (cm)		0.7 (0.3, 5.0)	0.9 (0.3, 3.1)	0.694^a^
Calculus type				0.389^b^
Non-urate stones		35 (87.5%)	20 (100%)	
Urate stones		1 (2.5%)	0 (0%)	
Mixed stones		4 (10.0%)	0 (0%)	
Hemoglobin (g/L)	130–175	138 (77, 168)	140 (101, 159)	0.754^a^
White Blood Cell (*10^9^/L)	3.5–9.5	7.24 (1.07, 19.40)	7.11 (5.11, 14.70)	0.683^a^
C-reactive protein (mg/L)	< 8.2	4.25 (0.50, 142.00)	2.68 (0.53, 104.00)	0.083^a^
Platelet (*10^9^/L)	125–350	236 (92, 524)	251 (100, 332)	0.644^a^
Neutrophils%	40–75	66.4 (±11.6)	70.2 (±10.0)	0.200^d^
Lymphocyte%	20–50	25.4 (±10.2)	22.1 (±8.8)	0.192^d^
Monocyte%	3–10	5.89 (±2.31)	5.58 (±1.84)	0.570^d^
Creatinine (umol/L)	M: 58–110, F: 49–92	83.2 (34.3, 181.0)	76.1 (42.1, 437.0)	0.567^a^
Urea (mmol/L)	M: 3.2–7.1, F: 2.5–6.1	6.04 (3.88, 13.10)	6.55 (3.89, 18.60)	0.583^a^
Uric acid (umol/L)	M: 208–428, F: 155–357	381 (±86)	374 (±116)	0.795^d^
Leukocyte esterase				0.103^a^
Negative		26 (65.0%)	9 (45.0%)	
Positive		12 (30.0%)	8 (40.0%)	
Strongly positive		2 (5.0%)	3 (15.0%)	
Nitrite				0.205^a^
Negative		39 (97.5%)	18 (90.0%)	
Positive		1 (2.5%)	1 (5.0%)	
Strongly positive		0 (0%)	1 (5.0%)	
PH	5.5–6.5	6.25 (5.00, 7.50)	6.00 (5.00, 7.50)	0.278^a^
Leukocyte microscopy	0–5	8 (0, 372)	18 (0, 199)	0.383^a^
Erythrocyte microscopy	0–4.5	14 (0, 34300)	21 (0, 30000)	0.838^a^

Values are number (percentage), median (Min, Max) or Mean ± SD.

^a^Calculated using the Wilcoxon rank-sum test.

^b^Calculated using the Fisher's exact test.

^c^Calculated using the chi-square test.

^d^Calculated using the independent Student's t-test.

Comprehensive clinical profiling revealed that routine serological and urinalysis parameters were highly comparable between the male and female cohorts. Specifically, no significant sex-based variances were observed in standard hematological indices (such as hemoglobin, white blood cell counts, platelet counts, and the percentages of neutrophils and lymphocytes) or in the acute-phase reactant C-reactive protein (CRP) (all P > 0.05). Similarly, key renal functional and routine biochemical parameters, namely serum urea, creatinine, and uric acid, remained consistent across both groups. Furthermore, detailed urinalysis, encompassing chemical evaluations (pH, leukocyte esterase, and nitrite) and microscopic urinary sediment analysis (WBC and red blood cell counts), demonstrated no significant differences. Crucially, the median and mean values of these routine hematological, renal, and urinary parameters for both sexes predominantly resided within normal physiological limits, effectively ruling out acute systemic inflammatory responses or severe metabolic derangements. Furthermore, the strict statistical comparability of these baseline variables ensures a symmetrical distribution of potential confounders between the sexes, thereby minimizing their capacity to independently drive the downstream immunological and microbial composition variations.

Despite this strict clinical uniformity in baseline metabolic and physiological parameters, peripheral blood cytokine profiling unveiled a pronounced sex-associated inflammatory profile. As shown in **Table 2**, the systemic immunological milieu of the male cohort was characterized by significantly elevated levels of specific pro-inflammatory cytokines, including IL-5, IL-17A, IFN-α, IL-12P70, and IFN-γ, compared to their female counterparts (P < 0.05). Crucially, when these profiles were contextualized against established clinical reference ranges, a nuanced pathophysiological pattern emerged. Both cohorts exhibited baseline elevations in several inflammatory markers (such as IL-6, IL-8, and TNF-α), reflecting the inherent inflammatory burden associated with active urolithiasis. However, male patients exhibited a significantly amplified, hyperactive immune state. Most notably, the median concentration of IL-17A—a key effector cytokine of the Th17 axis—breached the normal upper physiological threshold exclusively in the male cohort, while remaining robustly within normal limits in the female cohort. The remaining evaluated inflammatory factors exhibited no statistically significant disparities between the sexes.

**Table 2:** Inflammatory cytokine profiles in patients with urolithiasis stratified by sex.

Inflammatory factor (pg/ml)	Reference range	Male	Female	P. value^a^
		(N=40)	(N=20)	
IL2	< 5.71	1.53 (0.15, 4.76)	1.29 (0.27, 6.00)	0.262
IL4	< 2.80	1.97 (0.01, 10.03)	1.73 (0.01, 7.73)	0.180
IL5	< 3.10	1.11 (0.35, 2.50)	0.715 (0.01, 2.04)	0.013*
IL6	< 5.30	15.9 (2.23, 2180.47)	7.14 (1.86, 2500.00)	0.089
IL8	< 20.6	80.3 (6.08, 2500.00)	65.1 (2.56, 2500.00)	0.293
IL1β	< 12.4	3.67 (0.22, 97.55)	1.85 (0.01, 59.89)	0.057
IL17A	< 20.6	28.8 (2.16, 339.54)	19.2 (0.26, 60.09)	0.030*
IL10	< 4.91	3.04 (1.21, 18.82)	2.35 (0.94, 289.05)	0.083
IFNα	< 8.50	2.41 (0.01, 11.76)	1.33 (0.01, 11.80)	0.007**
TNFα	< 4.60	10.6 (1.07, 171.70)	5.34 (0.79, 43.50)	0.190
IL12P70	< 3.40	3.09 (0.01, 11.16)	1.73 (0.13, 7.22)	0.003**
IFNγ	< 7.42	3.37 (0.43, 14.83)	1.97 (0.22, 5.60)	0.022*

Values are number (percentage), median (Min, Max) or Mean ± SD.

^a^Calculated using the Wilcoxon rank-sum test.

*P <0.05 and **P <0.01.

### 3.2 Sex-driven divergence in gut microbial architecture

**Figure 1:**
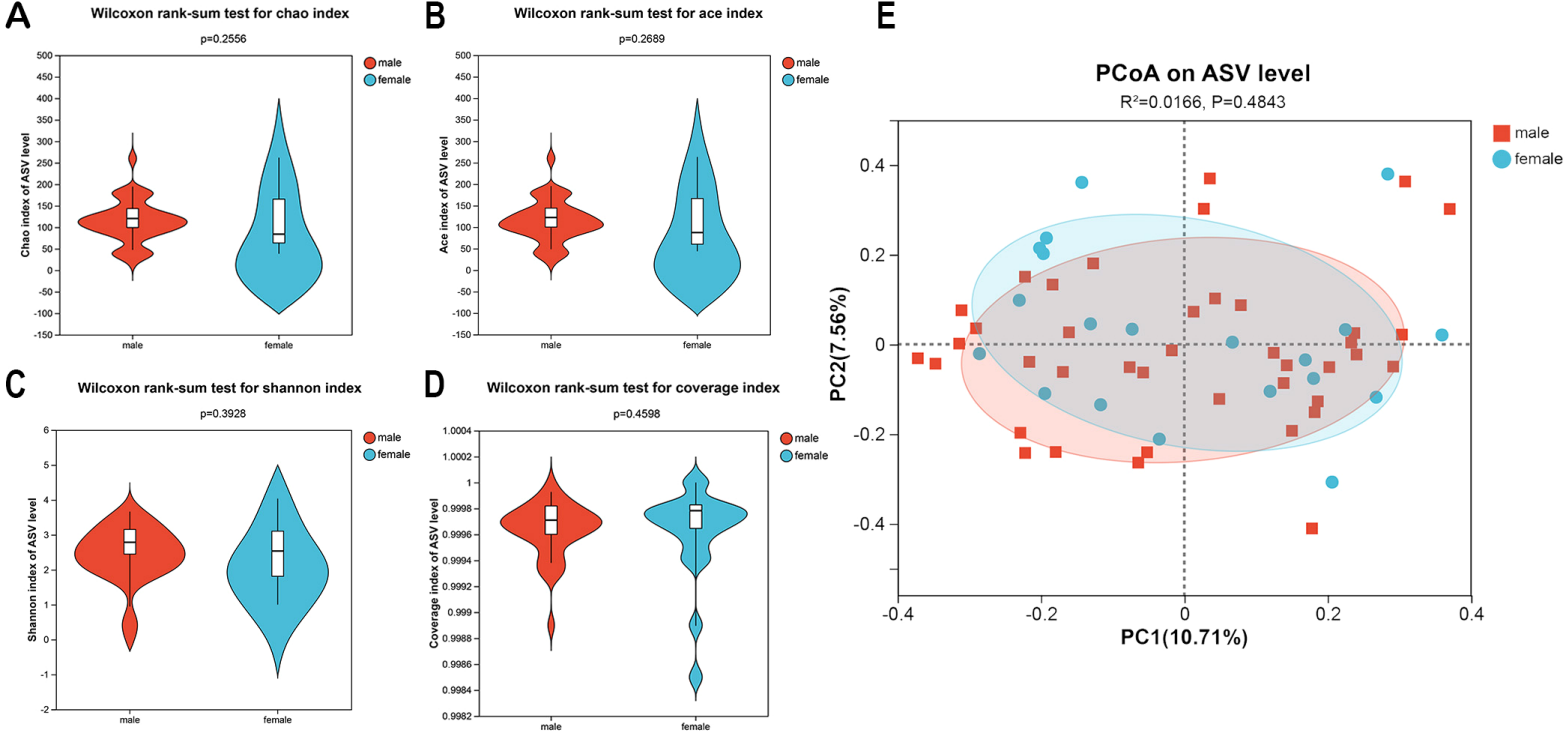
Gut microbiota diversity in male and female patients with urolithiasis. **(A–D)** Comparison of alpha-diversity indices (Chao1, ACE, Shannon, and Good’s coverage indices) at the ASV level between male and female patients (Wilcoxon rank-sum test). **(E)** Principal Coordinate Analysis (PCoA) based on the Bray-Curtis distance matrix at the ASV level. The Wilcoxon rank-sum test was utilized to assess structural differences along the primary principal coordinate axes (PC1 and PC2).

To delineate the variations in microbial community diversity between cohorts, 16S rRNA gene sequencing of fecal samples was conducted, revealing distinct bacterial structural compositions between male and female patients. Alpha-diversity evaluations at the ASV level, measured via Chao1, ACE, Shannon, and Good’s coverage indices, demonstrated no significant differences between the male and female groups (**Figures 1A–D**; all P > 0.05). Similarly, beta-diversity analysis via Principal Coordinate Analysis (PCoA) based on the Bray-Curtis distance matrix at the ASV level revealed no statistically significant difference in overall microbial community structure between the sexes (**Figure 1E**; Adonis R^2^ = 0.0166, P = 0.4843).

**Figure 2:**
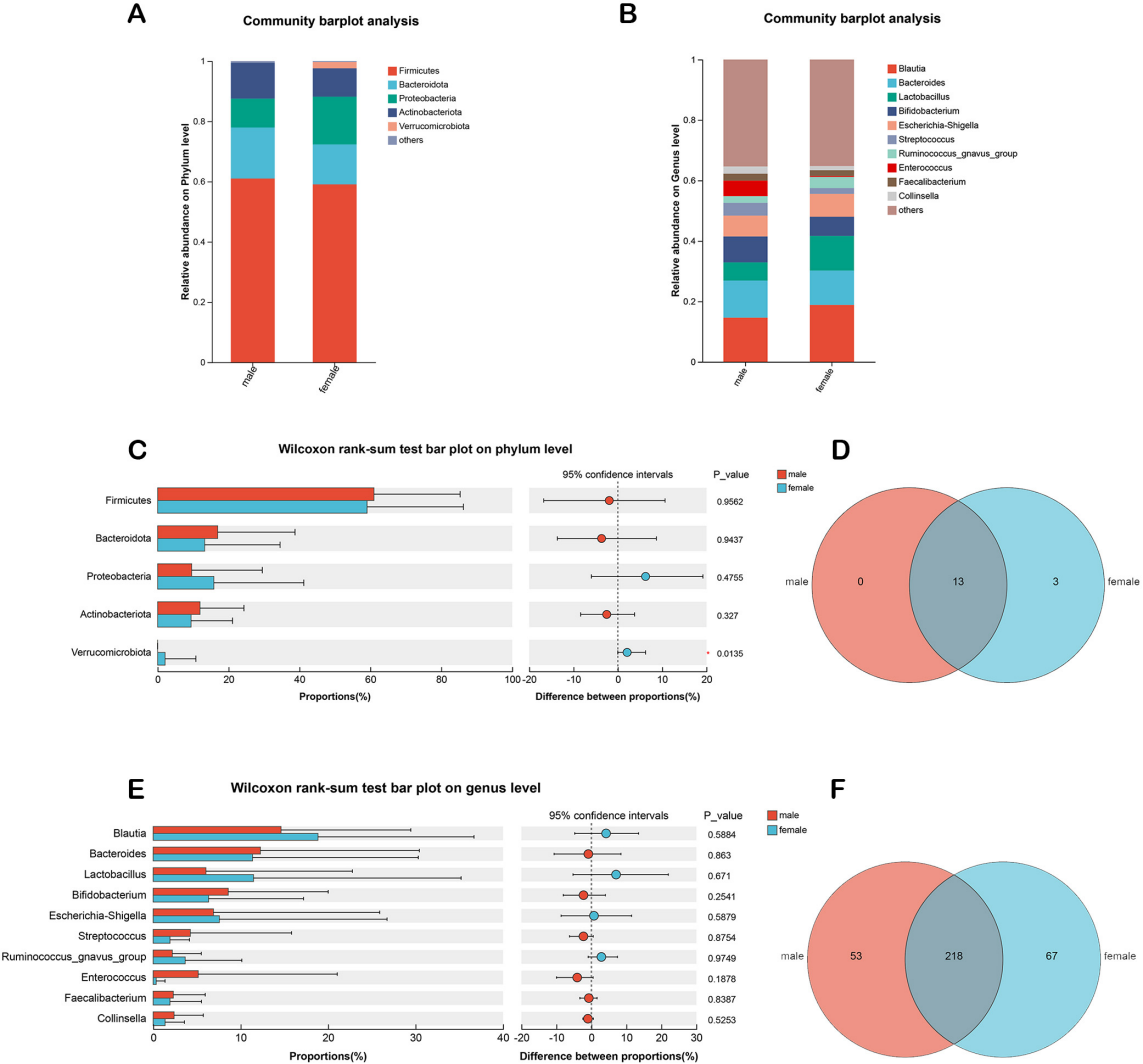
Sex-specific structural composition, differential abundance, and shared taxonomic features of the gut microbiota. **(A, B)** Relative abundance of dominant bacterial communities at the phylum **(A)** and genus **(B)** levels across male and female urolithiasis patients, presented as community barplots. **(C, E)** Wilcoxon rank-sum test bar plots demonstrating the differential proportions of the top phyla **(C)** and top 10 genera **(E)** between the sexes, accompanied by 95% confidence intervals and exact P values to explicitly illustrate inter-group variance. * P < 0.05. **(D, F)** Venn diagrams illustrating the distribution of shared and unique taxonomic units at the phylum **(D)** and genus **(F)** levels between the male and female cohorts.

Despite this macro-level structural similarity, to elucidate specific microscopic taxonomic shifts underlying the divergent clinical phenotypes, community compositions and the distribution of shared taxa were further interrogated at both the phylum and genus levels (**Figure 2**). At the phylum level, we evaluated the dominant phyla for each group (**Figure 2A**). The male cohort was primarily dominated by *Firmicutes* (61.00%), *Bacteroidota* (16.93%), and *Actinobacteriota* (11.94%), while the female cohort was dominated by *Firmicutes* (59.06%), *Proteobacteria* (15.85%), and *Bacteroidota* (13.26%). To explicitly address the variance associated with these taxonomic distributions, we performed Wilcoxon rank-sum tests with 95% confidence intervals (**Figure 2C**). This differential analysis revealed that despite the visual similarities in top-tier averages, the phylum *Verrucomicrobiota* was significantly enriched in the female cohort (P = 0.0135). An evaluation of shared taxonomic units further revealed that the two groups shared 13 ubiquitous phyla, with 3 phyla uniquely present in the female cohort and none exclusively found in males (**Figure 2D**).

Furthermore, high-resolution profiling of the dominant taxa at the genus level (**Figure 2B**) unveiled a more pronounced distributional divergence. Specifically, the male cohort was predominantly colonized by *Blautia* (14.62%), *Bacteroides* (12.26%), *Bifidobacterium* (8.58%), *Escherichia-Shigella* (6.89%), and *Lactobacillus* (6.03%). In contrast, the top five genera in females were *Blautia* (18.84%), *Lactobacillus* (11.47%), *Bacteroides* (11.36%), *Escherichia-Shigella* (7.56%), and *unclassified Enterobacteriaceae* (6.80%). Differential abundance analysis of the top 10 genera revealed no statistically significant differences in relative proportions between the male and female cohorts (**Figure 2E**). Following this, Venn diagram analysis at the genus level (**Figure 2F**) identified 218 core operational genera shared between both groups, alongside 53 unique genera in the male cohort (accounting for 15.68% of total assigned genera) and 67 unique genera in the female cohort (19.82%).

### 3.3 Identification of sex-specific discriminative taxa and their immune-metabolic associations

**Figure 3:**
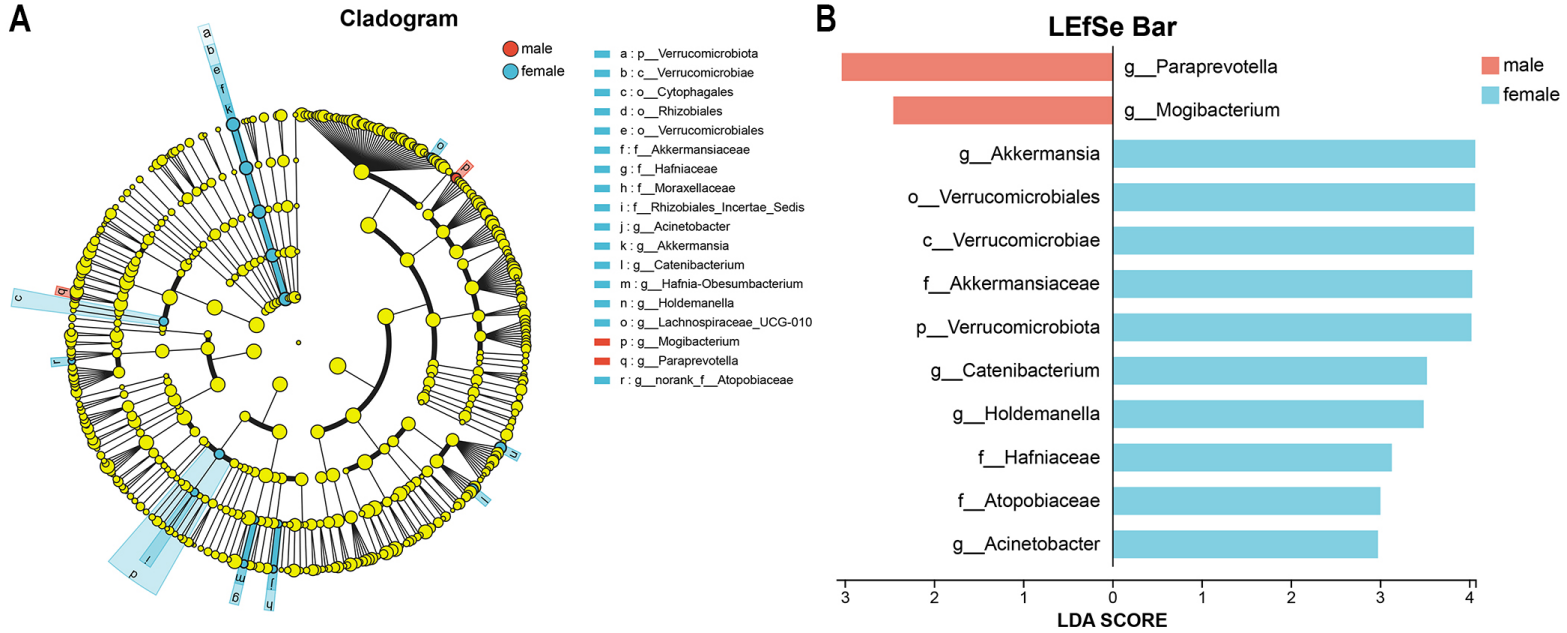
Identification of sex-specific differentially abundant taxa in urolithiasis patients using LEfSe analysis. **(A)** Taxonomic cladogram illustrating the phylogenetic distribution of differentially abundant microbiota between male and female patients. **(B)** Bar chart of Linear Discriminant Analysis (LDA) scores, showing the effect size of taxa significantly enriched in males or females (LDA score > 2.0, P < 0.05).

To identify the top discriminative taxa associated with the community divergence between sexes, Linear Discriminant Analysis Effect Size (LEfSe) was executed spanning the phylum to genus levels (LDA score > 2.0, P < 0.05). The analysis revealed a significant enrichment of the phylum *Verrucomicrobiota* and its subordinate taxa (class *Verrucomicrobiae*, order *Verrucomicrobiales*, family *Akkermansiaceae*, and genus *Akkermansia*) in the female cohort (**Figure 3A**). Notably, *Akkermansia* yielded the highest discriminative score (LDA = 4.07), marking it as the strongest microbial biomarker associated with the female phenotype (**Figure 3B**). Additionally, the genera *Catenibacterium* (LDA = 3.53), *Holdemanella* (LDA = 3.49), and *Acinetobacter* (LDA = 2.98) were significantly elevated in females. Conversely, the male cohort was characterized by a pronounced enrichment of *Mogibacterium* (phylum *Firmicutes*; LDA = 2.45) and *Paraprevotella* (phylum *Bacteroidota*; LDA = 3.03).

**Figure 4:**
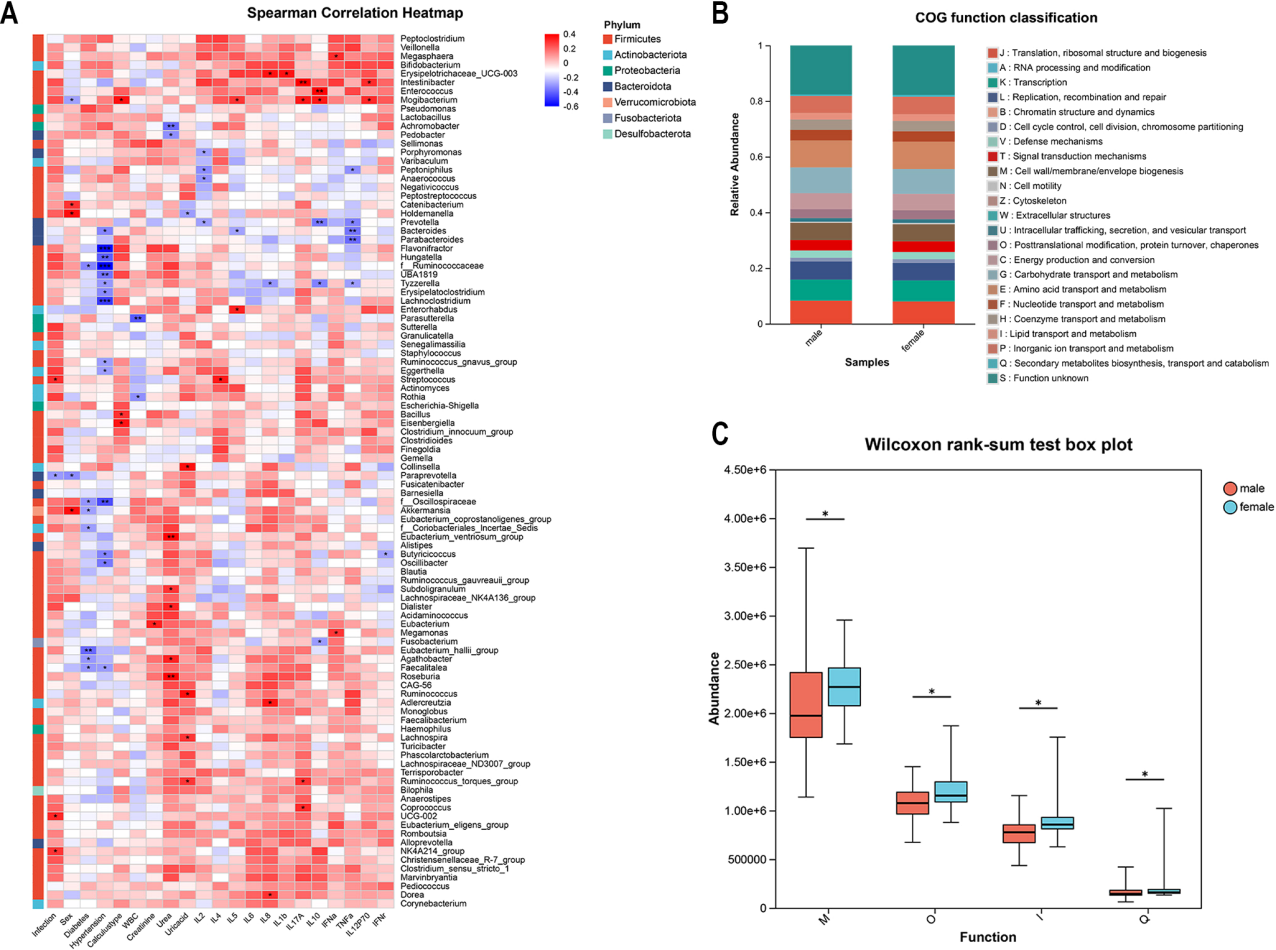
Correlation of discriminative microbiota with continuous clinical parameters and predicted functional profiles. **(A)** Spearman rank correlation heatmap illustrating the associations between the sex-specific discriminative bacterial genera and continuous clinical laboratory parameters (inflammatory cytokines and routine biochemical parameters). The heatmap incorporates the top 100 most abundant genera across the cohort, with unclassified taxa excluded. Hierarchical clustering for both taxonomic units and clinical indicators was executed using the average linkage method. **(B)** Mean relative abundance (percentages) of predicted COG (Clusters of Orthologous Groups) functional pathways in male and female patients, analyzed by PICRUSt2. **(C)** Box plots presenting the differentially abundant COG pathways between male and female cohorts based on their absolute predicted abundance values, evaluated by the Wilcoxon rank-sum test. *P < 0.05.

To evaluate the statistical correlations between the identified sex-specific discriminative genera and host physiological parameters, a preliminary Spearman’s rank correlation analysis was performed using raw (uncorrected) P-values. As illustrated in **Figure 4A**, this analysis focused exclusively on continuous clinical parameters, including systemic inflammatory cytokines and routine biochemical markers. Remarkably, *Mogibacterium*, a genus significantly enriched in male patients, exhibited robust positive synergies with multiple circulating pro-inflammatory and immunomodulatory cytokines. Specifically, its relative abundance was significantly and positively correlated with IL-5 (r=0.26, P<0.05), IL-17A (r=0.30, P<0.05), IL-12P70 (r=0.28, P<0.05), and IL-10 (r=0.31, P<0.05). Conversely, within the female cohort, the abundance of *Holdemanella* showed a significant negative correlation with serum uric acid levels (r=−0.27, P<0.05). No other significant linear correlations were observed between the remaining discriminative genera and the evaluated continuous laboratory parameters.

To evaluate the potential metabolic implications of these microbial shifts, the functional potential of the microbiome was inferred utilizing PICRUSt2, successfully mapping 23 predicted Clusters of Orthologous Groups (COG) pathways. While the overarching relative abundances (percentages) of these global pathways appeared visually comparable between the cohorts (**Figure 4B**), statistical comparisons based on their absolute predicted abundance values revealed significant functional divergences (**Figure 4C**). Specifically, the female cohort possessed significantly higher absolute predicted abundances within several core metabolic pathways compared to males. These upregulated functions specifically included: [Q] Secondary metabolites biosynthesis, transport, and catabolism (P = 0.049); [I] Lipid transport and metabolism (P = 0.015); [O] Posttranslational modification, protein turnover, chaperones (P = 0.021); and [M] Cell wall/membrane/envelope biogenesis (P = 0.038). The remaining predicted COG pathways exhibited no significant disparities between the two groups.

## 4 Discussion

The present study systematically evaluated the intra-cohort heterogeneity among clinically diagnosed urolithiasis patients. Our findings reveal that, while male and female patients share a highly comparable macro-level intestinal microbial architecture, their systemic immune milieu and specific core microbial signatures exhibit profound sex-associated divergence. Male patients demonstrated significantly elevated peripheral levels of pro-inflammatory cytokines, including IL-5, IL-17A, and IL-12P70, accompanied by a marked enrichment of the opportunistic intestinal pathogen *Mogibacterium*. Conversely, even in the diseased state, the female cohort retained a significant enrichment of beneficial commensals, predominantly *Akkermansia* and *Holdemanella*, alongside enhanced predicted metabolic capacities in lipid metabolism and secondary metabolite biosynthesis. These observations suggest that sex-specific variations in the gut microbiota and systemic inflammatory networks are closely associated with the profound pathophysiological heterogeneity within urolithiasis cohorts. Previous population-based studies indicate that even in healthy individuals, biological sex intrinsically shapes gut microbiome composition, with healthy females generally exhibiting stronger immune tolerance and distinct beneficial microbial profiles (Kim et al., 2020). Our results suggest that this baseline sex-driven divergence is closely associated with distinct microenvironmental patterns within the active urolithiasis state, with male patients tending to exhibit an amplified pro-inflammatory milieu, whereas female patients maintain a relatively resilient ecological profile.

The relatively attenuated systemic inflammatory phenotype observed in female patients is highly concurrent with the enrichment of specific commensals that are documented to modulate host metabolism and barrier function. Previous literature has established that *Akkermansia* reinforces the intestinal physical barrier via mucin degradation and modulates host lipid metabolism (Everard et al., 2013; Shaheen et al., 2025). Although our observational data cannot confirm causal pathways, we speculate based on these previous findings that its production of short-chain fatty acids (SCFAs) may be instrumental in regulating systemic immunity and potentially mitigating calcium oxalate stone formation (Jin et al., 2021). Concurrently, other experimental studies indicate that *Holdemanella* participates in uric acid metabolism and may exert potent anti-inflammatory effects through the synergistic secretion of long- and short-chain fatty acids (Pujo et al., 2021; Qie et al., 2025; Wang et al., 2026). The analysis of microbial functional potential further corroborates these microecological advantages: the significant upregulation of predicted pathways related to secondary metabolite biosynthesis (COG Q), lipid transport and metabolism (COG I), posttranslational modification and protein turnover (COG O), and cell wall/membrane biogenesis (COG M) in females implies a greater ecological resilience. Based on these functional predictions, we hypothesize that this resilience might provide the female gut microbiota with enhanced capacities for competitive inhibition of pathogen colonization (Deng and Wang, 2024), host lipid homeostasis modulation (Liu et al., 2026), and bacterial envelope fortification (Di Mattia et al., 2025; Di Vincenzo et al., 2024). However, these 16S-derived predictions require future validation via shotgun metagenomics and targeted metabolomics.

In stark contrast, the highly pro-inflammatory phenotype characterizing male urolithiasis patients coincides with a significantly higher relative abundance of opportunistic pathogens within their gut microbiota. This study demonstrates a robust positive correlation between the elevated abundance of *Mogibacterium* in males and core pro-inflammatory cytokines, notably IL-17A and IL-12P70. *Mogibacterium*, an opportunistic pathogen residing in the oral and intestinal microbiomes, is frequently enriched in various inflammatory disease models (Hale et al., 2017). Within the immune cascade, IL-12P70 drives Th1 cell differentiation and the subsequent hypersecretion of IFN-γ (Trinchieri, 2003; Singhto et al., 2018), whereas IL-17A serves as the primary effector cytokine of the Th17 response (Krebs et al., 2021); collectively, these patterns highlight a systemic Th1/Th17 pro-inflammatory phenotype in males. Based on existing literature, when these circulating pro-inflammatory cytokines infiltrate the local renal microenvironment (Mills, 2023), they are hypothesized to promote macrophage polarization and tubular epithelial stress, potentially contributing to the higher adhesive susceptibility of the urothelium to calcium oxalate crystals (Cao et al., 2015; Khan et al., 2021; Yuan et al., 2023). Furthermore, the significant elevation of IL-5 in the male cohort may reflect a chronic tissue remodeling process within the urinary tract driven by the persistent physical irritation of crystals (Gieseck et al., 2018). This sustained systemic pro-inflammatory state, accompanied by the enrichment of specific opportunistic gut pathogens, may represent a specific immunophenotype in male urolithiasis patients. Whether this profile precedes stone formation as a predisposing niche, or develops consequentially, warrants further investigation to understand its role in their higher clinical recurrence rates.

Notably, although the overall levels of the anti-inflammatory cytokine IL-10 did not significantly differ between sexes, its pronounced positive correlation with *Mogibacterium* suggests a compensatory negative feedback mechanism aimed at preserving immune homeostasis amid chronic inflammation (Saraiva and O'Garra, 2010). Conversely, the elevation of IFN-α, which lacked significant correlations with the identified differential taxa in our current analysis, implies that sex-specific systemic inflammation may also be compounded by other factors, such as sex hormones. Furthermore, the SCFA-producing protective commensal *Paraprevotella* (Fei et al., 2019) was paradoxically enriched in the systemically pro-inflammatory male cohort. This phenomenon may represent a compensatory community remodeling effort by the gut microbiome to restore intestinal homeostasis (Sommer et al., 2017; Parada Venegas et al., 2019).

This study holds significant theoretical value. By integrating multidimensional data, we propose a hypothesis that the sex disparity in urolithiasis may be closely linked to the potential crosstalk between opportunistic gut pathogens and the host’s immune-metabolic network. Moreover, the 2:1 male-to-female ratio of our cohort closely mirrors real-world epidemiological demographics, ensuring robust clinical generalizability. Several limitations warrant consideration. First, the cross-sectional design and lack of a healthy control group preclude establishing causality or determining whether these microecological signatures are antecedent triggers or disease consequences. Second, our female cohort encapsulated both postmenopausal and reproductive-aged individuals. Although we strictly standardized the biological sampling of premenopausal women to the early follicular phase to minimize cyclical hormonal confounding, the intrinsic differences in baseline immune tone between pre- and post-menopausal states may still restrict the absolute generalizability of the described female phenotype. Third, unquantified dietary variations between sexes may introduce confounding effects on the microbiome. Finally, 16S rRNA sequencing provides limited species-level resolution, and functional inference tools like PICRUSt2 yield only predicted functional potentials rather than direct measurements of true metabolic activity. Future longitudinal studies integrating strictly controlled diets, metagenomic sequencing, and metabolomics are essential to validate these findings and explore their utility in precision therapeutics.

In conclusion, our study highlights significant sex-associated differences in gut microecology and systemic immune microenvironments within urolithiasis patients. The male phenotype is characterized by *Mogibacterium* enrichment and a robust Th1/Th17 pro-inflammatory axis, whereas females harbor beneficial commensals (e.g., *Akkermansia* and *Holdemanella*) that confer metabolic and immune resilience. These sex-associated differences challenge the traditional ‘one-size-fits-all’ paradigm in urolithiasis management. Specifically, future strategies for mitigating male recurrence may benefit from integrated inflammatory evaluations and microbiome-modulating approaches, pending prospective clinical validation.

## Data Availability

The datasets presented in this study can be found in online repositories. The names of the repository/repositories and accession number(s) can be found below: https://www.ncbi.nlm.nih.gov/, SUB14390218.
